# HEALTH PROFILES OF OLDER NURSING HOME RESIDENTS BY SUICIDAL IDEATION: A LATENT CLASS ANALYSIS

**DOI:** 10.1093/geroni/igac059.2586

**Published:** 2022-12-20

**Authors:** Yiyang Yuan, Adrita Barooah, Deborah Mack, Kate Lapane, Christine Ulbricht

**Affiliations:** University of Massachusetts Chan Medical School, Burlington, Massachusetts, United States; University of Massachusetts Boston, Boston, Massachusetts, United States; University of Massachusetts Chan Medical School, Worcester, Massachusetts, United States; University of Massachusetts Chan Medical School, Worcester, Massachusetts, United States; National Institute of Mental Health, National Institutes of Health, Rockville, Maryland, United States

## Abstract

In US nursing homes, 2% of residents have documentation of suicidal ideation (SI). Whether older residents’ health profiles differ by SI is unknown. Using the 9th Patient Health Questionnaire-9 (PHQ-9) item on Minimum Data Set 3.0, we identified 15,277 older residents with and 562,184 without SI. Latent class analysis using frailty, cognitive impairment, palliative care index, pain, and remaining PHQ-9 items as indicators identified health profiles by at-admission SI and estimated the association between profiles and SI at 90 days. In residents with at-admission SI, four profiles emerged: (1) frail, intact/moderate cognitive impairment, all depressive symptoms (prevalence: 22.8%); (2) frail, moderate/severe pain, depressed mood, sleep problems, fatigue (32.2%); (3) frail, severe cognitive impairment, depressed mood, fatigue, feelings of worthlessness (22.9%); (4) pre-frail, moderate/severe cognitive impairment, depressed mood (22.0%). Compared to the residents in profile 4, those in profile 1 [adjusted OR (aOR): 1.24; 95% Confidence Interval (CI):1.11-1.37] and those in profile 2 [aOR: 1.11; 95% CI: 1.01-1.22] were more likely to continue reporting SI at 90 days. In residents without at-admission SI, three profiles emerged: (1) frail, depressed mood, fatigue (33.9%); (2) frail, severe cognitive impairment (38.1%); (3) prefrail/frail (28.0%). Residents in profile 1 were more [aOR: 2.80; 95% CI: 2.60-3.00] while those in profile 2 were less [aOR: 0.79; 95% CI: 0.71-0.86] likely profile 1 residents to report SI at 90 days. Findings indicate substantial heterogeneity in the health profiles between and within older residents with and without self-reported SI.

